# Rearrangements of Blood and Tissue Fatty Acid Profile in Colorectal Cancer - Molecular Mechanism and Diagnostic Potential

**DOI:** 10.3389/fonc.2021.689701

**Published:** 2021-05-27

**Authors:** Adriana Mika, Katarzyna Duzowska, Lukasz P. Halinski, Alicja Pakiet, Aleksandra Czumaj, Olga Rostkowska, Malgorzata Dobrzycka, Jaroslaw Kobiela, Tomasz Sledzinski

**Affiliations:** ^1^ Department of Pharmaceutical Biochemistry, Faculty of Pharmacy, Medical University of Gdansk, Gdansk, Poland; ^2^ Department of Environmental Analysis, Faculty of Chemistry, University of Gdansk, Gdansk, Poland; ^3^ Department of General, Endocrine and Transplant Surgery, Faculty of Medicine, Medical University of Gdansk, Gdansk, Poland

**Keywords:** colorectal cancer, gas chromatography-mass spectrometry, fatty acid, lipids, PCA, PLS-DA

## Abstract

Colorectal cancer (CRC) is often diagnosed at an advanced stage due to the invasiveness of colonoscopy; thus, non-invasive CRC diagnostics are desirable. CRC is associated with lipid alterations. We aimed to verify whether fatty acid (FA) profiles in CRC patients may serve as a potential diagnostic tool for CRC diagnosis. FA profiles were assayed by GC-MS in cancer tissue, paired normal mucosa and serum from CRC patients and healthy controls. The levels of very long FAs – VLCFAs (26:0, 28:0 and 26:1) were the most highly increased FAs in cancer tissue compared to normal colon mucosa. Moreover, these FA were present in serum of CRC patients, they were absent in the serum of healthy subjects, or present in only trace amounts. To verify if cancer cells are the source of small amounts of these VLCFAs in the serum of patients we performed experiment in HT-29 CRC cells, which proved that CRC cells can produce and release VLCFAs into the blood. Most importantly, we defined a panel of FAs that may be assayed in a single analysis that definitely distinguishes CRC patients and healthy subjects, which was confirmed by PLS-DA and multivariate ROC analysis (AUC = 0.985). This study shows that selected FA panel may serve as a diagnostic marker for CRC.

## Introduction

Colorectal cancer (CRC) is among the most common cancers occurring worldwide. Despite the well-known benefits of early detection, CRC is often diagnosed at an advanced stage (1). CRC screening is commonly performed; however, the invasiveness and discomfort of the most effective diagnostic method – colonoscopy – are high. This results in unsatisfactory screening compliance, and only one-third of eligible patients are diagnosed at an early stage (2). To obtain higher screening compliance and, consequently, a higher survival rate and lower cost of treatment, a minimally invasive serum-based test for risk stratification, detection and staging of CRC is needed (2).

Biochemically, the cancer tissue of CRC patients is characterized by alterations in lipid metabolism including alterations in fatty acid profiles (3, 4).

Fatty acids (FAs) display different metabolic effects (5). Recently, studies have shown that certain FAs or their metabolites could serve as diagnostic tools for CRC (6–8), but more research needs to be conducted to confirm these results. Some studies indicate that certain FA levels change depending on the stage of disease (5). Recently, our study confirmed these observations and showed other alterations in the FA profile in colorectal cancer tissue (9). Fatty acid profile alterations, such as the presence of cerotic acid (6) and decrease in the concentration of hydroxylated, polyunsaturated ultra-long-chain fatty acids (10), can also be found in the sera of CRC patients.

To the best of our knowledge, few studies have analyzed the correlation between CRC stages and whole FA profiles (5, 11, 12). In this study, we performed an analysis of the whole FA profile obtained from the sera of CRC patients as well as CRC tissue using gas chromatography paired with mass spectrometry and analyzed the results to examine whether the whole FA profile may serve as a diagnostic tool for the detection of CRC. Moreover, to verify the hypothesis that CRC cells are a source of very long-chain FAs - VLCFAs (26:0, 26:1, and 28:0) that are found exclusively in patient sera, an experiment using HT-29 CRC cell cultures was performed. Advanced statistical methods, including principal component analysis (PCA), were used to analyze the data set (13).

## Materials and Methods

### Patients

In this study, tissue samples from 92 CRC patients were included. Among them, we selected a subgroup of 44 CRC patients who were similar in terms of age to 35 healthy controls for the comparison of serum FA profiles in these two groups. The patients with stage I to IV CRC were treated with surgical resection of the large bowel segment. None of the patients received preoperative neoadjuvant treatment. The biochemical and clinical characteristics of the study subjects are presented in **Tables 1** and **2**. Tissue samples were collected from the tumor and normal large intestinal mucosa within the resection margin that were at least 5 cm from the tumor interface. Normal and cancer tissue samples were obtained from the same patient. Each sample was divided into two parts. The part used for the FA analysis was frozen in liquid nitrogen immediately after collection and stored in aliquots at -80°C until analysis. The other part was used for histopathological examination with H&E staining that was performed to confirm the presence of cancer and normal mucosa. The protocol of the study was compliant with the Declaration of Helsinki of the World Medical Association and with approval from the Local Bioethics Committee at the Medical University of Gdansk (decision no. NKBN/487/2015). Written informed consent was obtained from all the patients prior to the study.

**Table 1:** Biochemical characteristics of the study subjects.

Parameter	HC	CRC1	CRC2	p (HC *vs* CRC1)	p (HC *vs* CRC2)
Sex	17m/18f	50m/42f	24m/20f	–	–
Age (years)	53.3 ± 1.60	68.6 ± 1.26	57.3 ± 1.17	<0.001	0.057
BMI (kg/m^2^)	26.6 ± 0.69	26.7 ± 0.59	26.8 ± 0.64	0.835	0.789
CRP-hs (mg/L)	1.90 ± 0.22	4.43 ± 0.64	3.58 ± 0.77	<0.001	<0.001
Total serum cholesterol (mg/dL)	204 ± 7.69	171 ± 6.02	189 ± 8.72	<0.001	0.008
Triacylglycerols (mg/dL)	129 ± 10.5	126 ± 75.1	140 ± 14.8	0.154	0.077
HDL (mg/dL)	54.9 ± 2.29	41.9 ± 1.27	42.5 ± 1.88	<0.001	<0.001
LDL (mg/dL)	123 ± 6.90	111 ± 4.61	126 ± 7.32	<0.001	<0.001
Glucose (mg/dL)	99.8 ± 3.98	112 ± 3.79	110 ± 4.16	0.028	0.065
Albumin (g/dL)	3.98 ± 0.44	3.34 ± 0.72	3.43 ± 1.11	<0.001	<0.001
Total protein (g/dL)	7.43 ± 0.083	6.51 ± 0.14	6.59 ± 0.21	<0.001	0.001

HC, healthy controls; CRC1, whole group of CRC patients; CRC2, subgroup of CRC patients, that do not differ in terms of age from HC group, from whom serum was analyzed. BMI, body mass index; CRP-hs – high-sensitivity C-reactive protein; HDL, high density lipoprotein; LDL, low density lipoprotein; SEM, standard error of the mean. Values are mean ± SEM.

**Table 2:** Clinical characteristics of the CRC patients.

Parameter	CRC1	CRC2
Location of primary tumor		
Cecum	19	11
Ascending colon	10	2
Transverse colon	11	3
Descending/proximal sigmoid colon	20	13
Cecum AND Descending/proximal sigmoid colon	1	0
Rectosigmoid	15	5
Rectum	16	10
T stage		
T1	5	3
T2	18	10
T3	57	25
T4	12	6
UICC stage		
I	18	10
II	27	14
III	33	12
IV	14	8
Lymph node status		
N0	48	26
N1/N2	44	18
Degree of differentiation		
Well differentiated	7	6
Moderately differentiated	75	34
Poorly differentiated	10	4

CRC1, whole group of CRC patients; CRC2 – subgroup of CRC patients, that do not differ in terms of age from healthy control group, from whom serum was analyzed. T stage, tumor stage; UICC stage, Union for International Cancer Control stage.

### CRC Cell Supplementation With ^13^C-18:0

The HT-29 cell line (human colon adenocarcinoma cells) was obtained from the American Tissue Culture Collection (ATCC) and cultured as described previously (14). An equal number of cells were seeded in 100 mm plates with standard medium or with medium + ^13^C-18:0 (75 μM) for 24 and 72 h. ^13^C-18:0 was dissolved in dimethyl sulfoxide (DMSO) and mixed with the standard medium 24 h before the experiment. Neither DMSO nor ^13^C-18:0 had any effect on cell viability. After incubation, the culture medium was collected. Cells were washed with phosphate-buffered saline (PBS), detached from the plates by a trypsin/EDTA solution (0.05%/0.02%), and centrifuged at 700 × g for 5 min at 8°C. After discarding the supernatant, the cell pellet was resolved in PBS. Cells and culture media were frozen in liquid nitrogen immediately after collection and stored at −20°C for further analysis.

### Fatty Acid Profile Analysis

Extraction of total lipids from tissue and serum samples was performed according to the method of Folch et al. (15). After lipid hydrolysis with KOH in methanol, washing with water/n-hexane, the n-hexane phase was evaporated to dryness under a stream of nitrogen. Free FAs were then methylated with 10% boron trifluoride. The FA methyl esters (FAMEs) were analyzed with GC-EI-MS QP-2010 SE (Shimadzu, Japan), as described by Mika et al. (14).

### SIM Analysis of the Products of the Conversion of ^13^C-Labelled Stearic Acid in HT-29 Cells

The extraction and hydrolysis of lipids from HT-29 CRC cells treated with ^13^C-18:0 and media and the derivatization of FAs into FAMEs were performed as mentioned above. Characteristic fragment ions were selected to identify product elongation and the desaturation or shortening of ^13^C-labelled stearate, including saturated FA (SFA) and monounsaturated FA (MUFA) from 14:0 to 32:0. We did not analyze the characteristic fragment ions of polyunsaturated FA (PUFA), since in human cells, PUFA cannot be produced from SFA or MUFA (16). The fragment ions were analyzed by GC-MS with selective ion monitoring (SIM). The overall run time of the analysis was 67.5 min, and the column temperature was set between 60-310°C. The chromatographic and mass conditions of GC-MS analysis remained the same. Least squares regression analysis was implemented by comparing the peak area ratios to the increasing standard concentrations to obtain calibration linearity.

### Statistical/Chemometric Analysis

The statistical significance of the differences in the study parameters was verified with a paired Student’s t-test (cancer tissue vs. normal colorectal mucosa) for data with a normal distribution, and a Wilcoxon signed-rank test was used for data with a non-normal distribution. The differences were considered significant at p < 0.05. The results are presented as the means ± standard error of the mean (SEM). All statistical calculations were carried out with SigmaPlot software (Systat, Software Inc., San Jose, CA, USA).

The chemometric data analysis was carried out using the computing environment R (17) (R Core Team). Two-way hierarchical cluster analysis (HCA) was carried out using the mixOmics package (18). Cluster analysis was performed using the Ward method, with the squared Euclidean distance used as the measure of similarity. Principal component analysis (PCA) was performed using the FactoMineR package (19) with the factoextra package for data visualization. All data matrices were autoscaled before the analysis. The PCA results were statistically processed using an unpaired t-test, and differences were accepted as statistically significant at p < 0.01. The PLS-DA models and ROC analysis were generated with the application MetaboAnalyst 4.0 (20).

## Results

### Serum Fatty Acid Profile

The results of the basic lipidogram analysis revealed dyslipidaemia in CRC patients, in contrast to healthy controls (HC) (**Table 1**). Due to the limited information obtained based on the lipoprotein fractions, we used another method to determine the lipidome of CRC patients. The more advanced analysis of the FA profile by mass spectrometry showed that the content of some individual FAs in the serum of HC and CRC patients was very different (**Table 3**). The total monounsaturated fatty acids (MUFAs) were increased in the serum of CRC patients, mostly due to the increased amount of 18:1. However, it should be noted that despite the strong significance of the difference in serum 18:1 between HC and CRC patients, this difference was not large – it was only approximately 8%.

**Table 3:** Fatty acids profile (%) in serum of healthy controls (HC) and CRC patients.

	HC n=35	CRC n=44	p
10:0	0.021 ± 0.001	0.019 ± 0.006	0.804
12:0	0.25 ± 0.019	0.14 ± 0.016	<0.001
14:0	1.20 ± 0.051	0.88 ± 0.059	<0.001
16:0	23.3 ± 0.29	23.3 ± 0.33	0.966
18:0	7.25 ± 0.12	6.88 ± 0.16	0.073
20:0	0.078 ± 0.004	0.088 ± 0.005	0.113
22:0	0.15 ± 0.008	0.17 ± 0.009	0.151
24:0	0.14 ± 0.006	0.14 ± 0.009	0.906
26:0	traces	0.020 ± 0.007	–
28:0	ND	traces	–
**Total ECFA**	**32.4 ± 0.31**	**31.7 ± 0.32**	**0.103**
11:0	0.013 ± 0.001	0.005 ± 0.001	<0.001
13:0	0.027 ± 0.002	0.012 ± 0.002	<0.001
15:0	0.23 ± 0.009	0.23 ± 0.010	0.768
17:0	0.25 ± 0.007	0.25 ± 0.008	0.954
19:0	0.031 ± 0.002	0.019 ± 0.002	<0.001
21:0	0.014 ± 0.001	0.013 ± 0.001	0.395
23:0	0.056 ± 0.003	0.050 ± 0.004	0.190
**Total OCFA**	**0.63 ± 0.017**	**0.58 ± 0.021**	**0.093**
13-methyl-14:0	0.031 ± 0.002	0.020 ± 0.002	<0.001
14-methyl-15:0	0.073 ± 0.003	0.054 ± 0.007	0.014
other iso-BCFA	0.13 ± 0.007	0.13 ± 0.008	0.717
**Total iso BCFA**	**0.24 ± 0.011**	**0.20 ± 0.014**	**0.063**
12-methyl-14:0	0.046 ± 0.002	0.032 ± 0.004	0.002
14-methyl-16:0	0.12 ± 0.007	0.067 ± 0.004	<0.001
20-methyl-22:0	0.003 ± 0.000	0.005 ± 0.001	0.061
**Total anteiso BCFA**	**0.17 ± 0.008**	**0.10 ± 0.008**	**<0.001**
**Total SFA**	**33.5 ± 0.30**	**32.6 ± 0.34**	**0.054**
14:1	0.075 ± 0.005	0.039 ± 0.004	<0.001
16:1	3.11 ± 0.16	3.02 ± 0.13	0.669
18:1	27.0 ± 0.50	29.2 ± 0.43	0.001
19:1	0.027 ± 0.002	0.021 ± 0.003	0.059
20:1	0.17 ± 0.007	0.16 ± 0.008	0.145
22:1	0.034 ± 0.004	0.021 ± 0.003	0.006
24:1	0.23 ± 0.017	0.24 ± 0.015	0.689
26:1	ND	0.001 ± 0.000	–
**Total MUFA**	**30.6 ± 0.57**	**32.7 ± 0.49**	**0.007**
CPOA2H	0.17 ± 0.005	0.11 ± 0.006	<0.001
ALA	0.34 ± 0.018	0.23 ± 0.017	<0.001
EPA	1.08 ± 0.13	0.87 ± 0.060	0.153
DHA	1.14 ± 0.076	1.43 ± 0.077	0.011
other PUFAn-3	0.39 ± 0.010	0.41 ± 0.016	0.301
**Total PUFAn-3**	**2.95 ± 0.20**	**2.94 ± 0.14**	**0.963**
LA	25.7 ± 0.61	24.2 ± 0.55	0.077
DGLA	1.16 ± 0.039	1.15 ± 0.056	0.821
ARA	5.60 ± 0.20	6.00 ± 0.211	0.175
AdA	0.10 ± 0.005	0.12 ± 0.014	0.134
other PUFAn-6	0.23 ± 0.008	0.19 ± 0.008	<0.001
**Total PUFAn-6**	**32.8 ± 0.63**	**31.7 ± 0.63**	**0.217**

Values are mean ± SEM. ND, not detected. AdA, adrenic acid (22:4 n-6); ALA, linolenic acid (18:3 n-3); ARA, arachidonic acid (20:4 n-6); BCFA, branched chain fatty acids; CPOA2H, cyclopropaneoctanoic acid 2-hexyl; DGLA, dihomo–linolenic acid (20:3 n-6); DHA, docosahexaenoic acid (22:6 n-3); DPA, docosapentaenoic acid (22:5 n-3); ECFA, even chain fatty acids; EPA, eicosapentaenoic acid (20:5 n-3); LA, linoleic acid (18:2 n-6); MUFA, monounsaturated fatty acids, OCFA, odd chain fatty acids; PUFA, polyunsaturated fatty acids; SFA, saturated fatty acids. Bold represents main groups of fatty acids.

Among the polyunsaturated fatty acids (PUFA) group, we observed significantly decreased alpha-linolenic acid (ALA) in the serum of CRC patients, and in the case of linoleic acid (LA), a downward trend was observed (**Table 3**). Among saturated fatty acids (SFAs) cerotic acid (26:0) was detected only in trace amounts in the HC group, unlike the CRC group, whereas montanic acid (28:0) was not observed in the HC group at the LOD or LOQ level of the analytical instrument used for GC-MS analysis and was detected in trace amounts in the serum of CRC patients (**Table 3**). Furthermore, the representative of VLCFA from the MUFA group – hexacodenoic acid (26:1) – was not detected in serum from HCs and was present in small amounts in CRC patients. In turn, FAs with shorter chains (11:0, 12:0, 13:0, and 14:0) were detected at levels in the serum of CRC patients that were several-fold lower than those in HCs, which is similar to the results for several representatives of branched chain FA (BCFAs) (**Table 3**).

### Fatty Acid Profile in Cancer Tissue and Normal Colon Mucosa

Then, we analyzed the FA profiles in the tumor tissue and paired normal colon mucosa of every CRC patient. We found significant differences between the normal mucosa and cancer tissue (**Table 4**). The levels of MUFA were significantly decreased; however, very long n-6 PUFAs were found at higher levels in cancer tissues than in normal mucosa. In turn, the levels of n-3 PUFAs were almost two-fold higher in cancer tissues (**Table 4**). BCFAs, regardless of the tissue, were at the same levels, whereas the levels of odd chain fatty acids (OCFAs) were elevated in cancer tissues (**Table 4**). In cancer tissue, we observed a large accumulation of both saturated and monounsaturated VLCFAs compared to that in normal mucosal tissue. The differences between the content of these VLCFAs in tumor comparing to normal tissue were the greatest and were up to 10-fold in the case of 26:1, so if cancer cells could release these VLCFAs outside the cell, one can expect that cancer cells are the source of small amounts of this VLCFA in the serum of patients. To verify this hypothesis, we performed an *in vitro* experiment using HT-29 CRC cells, which were treated with ^13^C-labelled stearate (^13^C-18:0).

**Table 4:** Profile of fatty acids (%) in normal mucosa and CRC tissues.

	NORMAL tissue n=92	CANCER tissue n=92	p
16:0	21.6 ± 0.20	20.7 ± 0.22	<0.001
18:0	7.78 ± 0.27	11.9 ± 0.35	<0.001
20:0	0.17 ± 0.008	0.28 ± 0.014	<0.001
22:0	0.15 ± 0.011	0.27 ± 0.015	<0.001
24:0	0.14 ± 0.012	0.38 ± 0.026	<0.001
26:0	0.010 ± 0.001	0.051 ± 0.006	<0.001
28:0	0.002 ± 0.000	0.009 ± 0.001	<0.001
other ECFA	2.57 ± 0.09	1.92 ± 0.09	<0.001
**Total ECFA**	**32.4 ± 0.33**	**35.4 ± 0.36**	**<0.001**
15:0	0.32 ± 0.010	0.33 ± 0.012	0.497
17:0	0.34 ± 0.013	0.40 ± 0.014	<0.001
19:0	0.023 ± 0.001	0.041 ± 0.002	<0.001
21:0	0.014 ± 0.001	0.022 ± 0.002	<0.001
23:0	0.033 ± 0.003	0.069 ± 0.005	<0.001
Other OCFA	0.022 ± 0.001	0.022 ± 0.001	0.618
**Total OCFA**	**0.75 ± 0.023**	**0.88 ± 0.026**	**<0.001**
iso BCFA	0.24 ± 0.008	0.24 ± 0.008	0.753
anteiso BCFA	0.15 ± 0.007	0.15 ± 0.007	0.785
**Total SFA**	**33.6 ± 0.35**	**36.7 ± 0.37**	**<0.001**
16:1	4.57 ± 0.17	3.70 ± 0.12	<0.001
18:1	42.9 ± 0.57	36.0 ± 0.65	<0.001
20:1	0.63 ± 0.022	0.72 ± 0.032	0.011
22:1	0.054 ± 0.004	0.113 ± 0.007	<0.001
24:1	0.15 ± 0.01	0.43 ± 0.032	<0.001
26:1	0.002 ± 0.000	0.020 ± 0.002	<0.001
other MUFA	0.22 ± 0.014	0.13 ± 0.009	<0.001
**Total MUFA**	**48.5 ± 0.68**	**41.1 ± 0.72**	**<0.001**
CPOA2H	0.19 ± 0.007	0.20 ± 0.010	<0.001
ALA	0.037 ± 0.002	0.049 ± 0.004	0.003
EPA	0.26 ± 0.020	0.45 ± 0.028	<0.001
DHA	0.60 ± 0.03	1.07 ± 0.05	<0.001
other PUFAn-3	0.37 ± 0.014	0.63 ± 0.028	<0.001
**Total PUFAn-3**	**1.27 ± 0.06**	**2.20 ± 0.10**	**<0.001**
LA	11.6 ± 0.21	10.9 ± 0.23	0.012
DGLA	0.34 ± 0.03	1.19 ± 0.058	<0.001
ARA	3.55 ± 0.24	6.40 ± 0.32	<0.001
AdA	0.40 ± 0.02	0.84 ± 0.056	<0.001
other PUFAn-6	0.39 ± 0.027	0.40 ± 0.02	0.564
**Total PUFAn-6**	**16.4 ± 0.43**	**19.8 ± 0.48**	**<0.001**

Values are mean ± SEM. AdA, adrenic acid (22:4 n-6); ALA, α-linolenic acid (18:3 n-3); ARA, arachidonic acid (20:4 n-6); BCFA, branched chain fatty acids; CPOA2H, cyclopropaneoctanoic acid 2-hexyl; DGLA, dihomo-γ-linolenic acid (20:3 n-6); DHA, docosahexaenoic acid (22:6 n-3); ECFA, even chain fatty acids; EPA, eicosapentaenoic acid (20:5 n-3); LA, linoleic acid (18:2 n-6); MUFA, monounsaturated fatty acids, OCFA, odd chain fatty acids; PUFA, polyunsaturated fatty acids; SFA, saturated fatty acids. Boldface - major groups of fatty acids.

### The Treatment of HT-29 CRC Cells With ^13^C-Labelled Stearate

The characteristic fragment ions of the labelled products were identified by single ion monitoring (SIM) analysis, and their content was determined based on internal standards.

**Figure 1:**
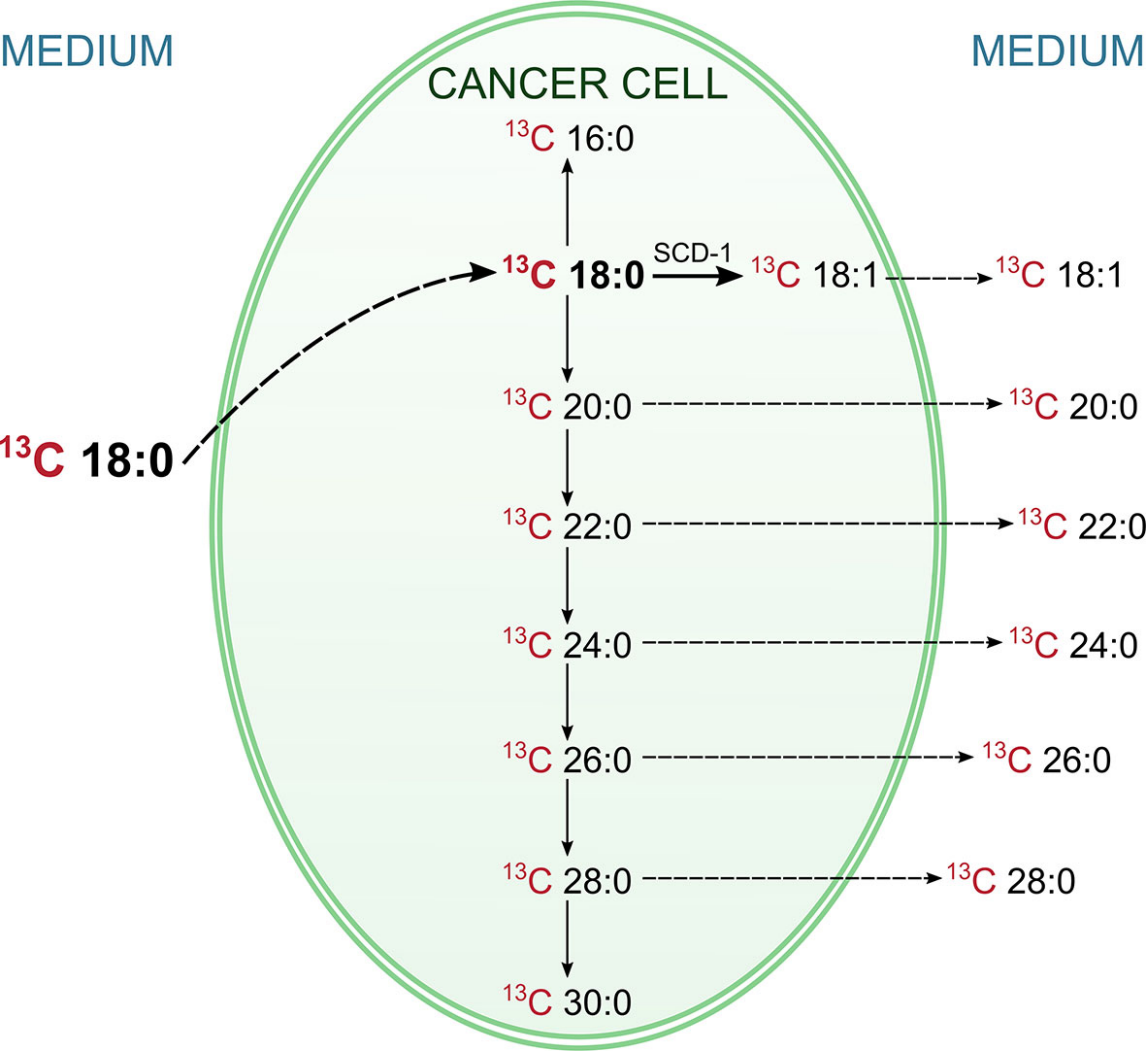
Pathways of conversions of labelled ^13^C stearic acid in HT-29 CRC cell. The labelled ^13^C stearic acid was added into HT-29 CRC cell culture, and during the incubation its level in medium was decreasing, whereas inside the cells was increasing. This indicate, that ^13^C stearate has been absorbed by cancer cells. At the same time the labeled products of the metabolism of this FA (including VLCFA and oleic acid) appeared in the cells. Moreover, they appeared also in the medium, and their levels in medium was increasing. This experiment proved that CRC cells can synthesize oleic acid and VLCFA from stearic acid and release them outside the cell.

As presented in **Table 5**, the level of ^13^C-labelled stearate in the medium during incubation decreased, which suggests that this FA is transported into cancer cells. This supposition is supported by the fact that ^13^C-18:0 appears inside HT-29 cells during incubation (**Table 5**). Moreover, after 24 h and 72 h of incubation, we were able to detect other ^13^C-FAs inside the cells, which means that ^13^C-18:0 is metabolized into other FAs. The major product of ^13^C-labelled stearate is 18:1, but we also found saturated VLCFAs with 20-30 carbons in their chains (**Table 5**), which suggests that the abovementioned FAs are the main products of 18:0 metabolism in CRC cells. Most importantly, we found most of these FAs in the culture medium, and their levels increased during incubation (**Table 5**). These results revealed that CRC cells used ^13^C-18:0 to produce VLCFAs and oleate, which were then released outside the cells into the medium. The other fatty acids mentioned in section *SIM Analysis of the Products of the Conversion of ^13^C-Labelled Stearic Acid in HT-29 Cells* were not identified, which indicates that ^13^C-18:0 is not metabolized into shorter SFAs (12:0, 14:0) and MUFAs (14:1, 16:1). The pathways of 18:0 conversion in CRC cells are summarized in **Figure 1**.

**Table 5:** The amounts of ^13^C labelled fatty acids (ng) in the media and HT29 CRC cells after culture.

		Fragment ions m/z	0h	24h	72h
MEDIUM	^13^C-18:0	76; 316	8210	456 ± 23.6	158 ± 14.4
	^13^C-20:0	76; 344	0	3.22 ± 0.37	6.68 ± 1.01
	^13^C-22:0	76; 372	0	2.26 ± 0.43	6.62 ± 1.89
	^13^C-24:0	76; 400	0	2.11 ± 0.33	17.9 ± 2.60
	^13^C-26:0	76; 428	0	1.47 ± 0.37	7.94 ± 2.16
	^13^C-28:0	76; 456	0	0.21 ± 0.04	0.41 ± 0.12
	^13^C-18:1	76; 314	0	13.5 ± 1.48	49.1 ± 6.98
HT-29 CELLS	^13^C-16:0	76; 288	0	0.92 ± 0.58	1.25 ± 0.08
	^13^C-18:0	76; 316	0	1433 ± 62.7	531 ± 13.9
	^13^C-20:0	76; 344	0	54.8 ± 3.65	28.6 ± 1.07
	^13^C-22:0	76; 372	0	28.2 ± 3.22	22.2 ± 1.27
	^13^C-24:0	76; 400	0	76.6 ± 11.71	70.5 ± 2.62
	^13^C-26:0	76; 428	0	30.2 ± 5.52	29.7 ± 1.49
	^13^C-28:0	76; 456	0	1.63 ± 0.30	1.57 ± 0.12
	^13^C-30:0	76; 484	0	traces	–
	^13^C-18:1	76; 314	0	336 ± 16.1	484 ± 5.07

Values are mean ± SEM.

### Examination of Diagnostic Potential of Fatty Acid Profile by Chemometric Methods

**Figure 2:**
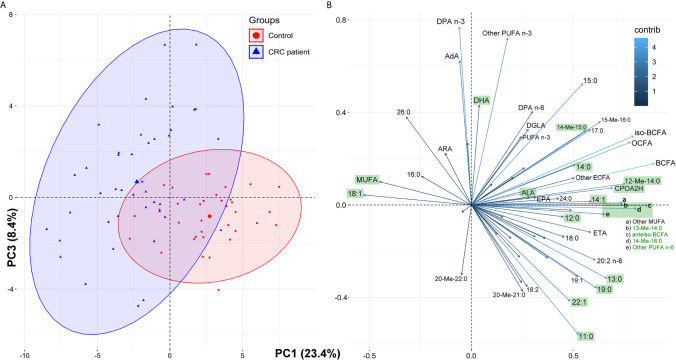
The results of principal component analysis (PCA) based on the whole fatty acids profile in serum of the study subjects: score plots of cases **(A)** and variables **(B)**. Fatty acids marked green displayed statistically significant differences in their average serum concentrations between the control group and CRC patients (paired t-test, p < 0.05; see **Table 1** for details and abbreviations); some minor variables were not shown to keep the clarity of presentation.

To examine whether the whole FA profile may potentially serve as a diagnostic tool for the detection of CRC, we performed multivariate analysis. The selection of the most appropriate principal components was based on differences in average PC values between patient groups (**Supplementary Table S1**). However, PCA showed that the whole FA profile in the serum of CRC patients was only partially different compared to that in the serum of healthy subjects (**Figure 2**). Therefore, the whole serum FA profile cannot serve as a standalone diagnostic tool.

**Figure 3:**
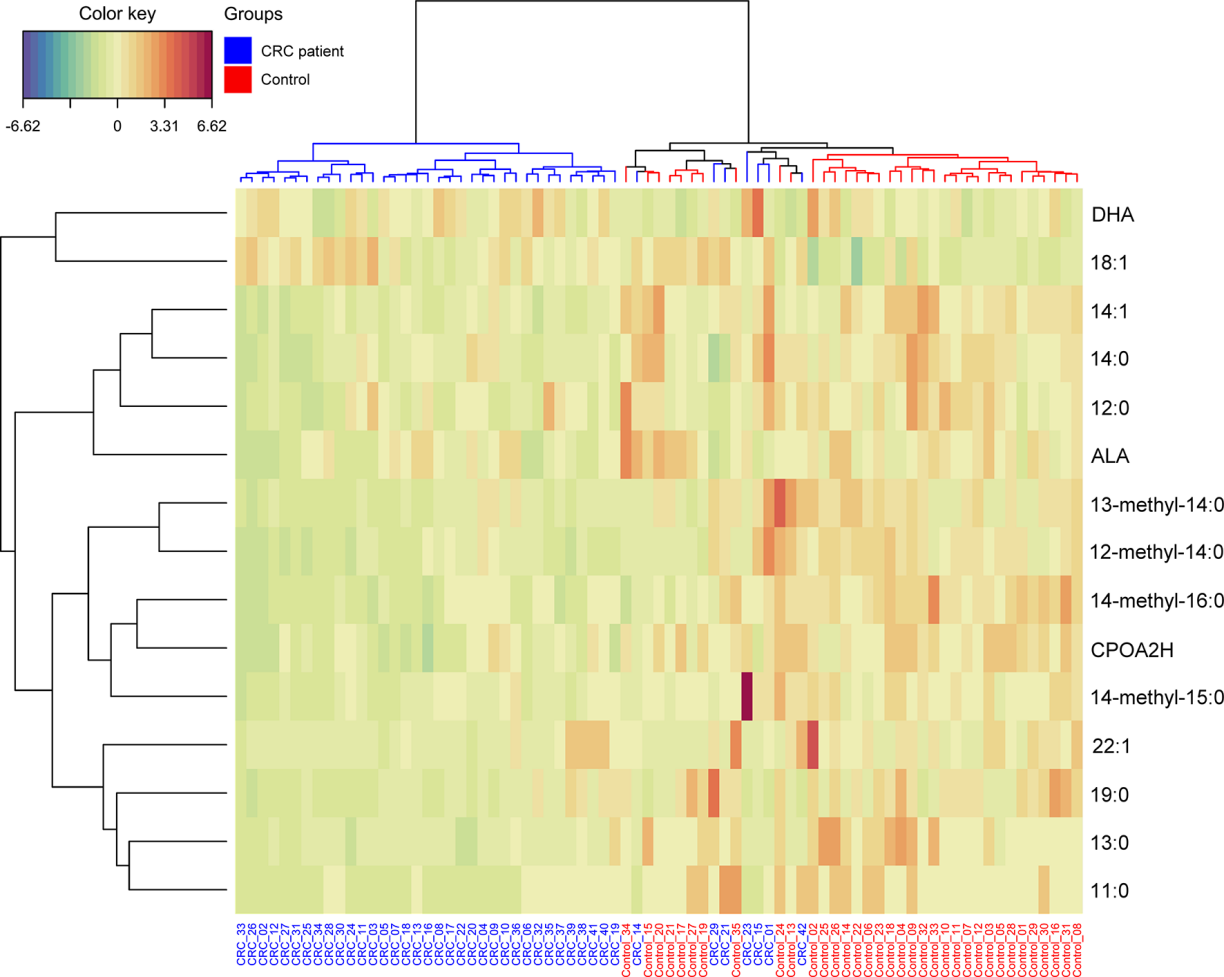
Heatmap representing the relative levels of fatty acids among two tested groups. Warm colors on the heatmap indicate high content of a given FA in relation to the mean value in the whole dataset, while cold colors represent its low content when compared to the average. The serum samples of CRC patients and HCs are represented by blue and red, respectively.

The most promising FA, that were significantly different between HC and CRC patient serum, were selected on the basis of the results in **Table 3**. These 15 FAs were visualized by a heatmap using two-way hierarchical cluster analysis (**Figure 3**). The serum samples of CRC patients and HCs are clustered separately. This separation suggests that the profiles of selected FA are quite different between the two research groups.

**Figure 4:**
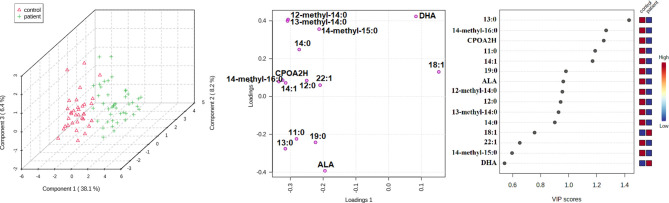
Partial least squares regression (PLS-DA) model, biplot and VIP score for comparison of selected FA in serum of CRC patients and healthy controls.

To examine whether multivariate analysis of the group of 15 selected FAs may potentially be used in diagnostics, a PLS-DA model was built (**Figure 4**). The VLCFAs 26:0, 28:0 and 26:1 were not included in this analysis because they were absent or present in only trace amounts in the sera of subjects from the HC group. An evident separation between these two groups can be easily observed for the first and second latent variables (LVs). The model was statistically significant at p < 0.05. Tridecanoic acid was identified as the FA with the highest coefficient score.

**Figure 5:**
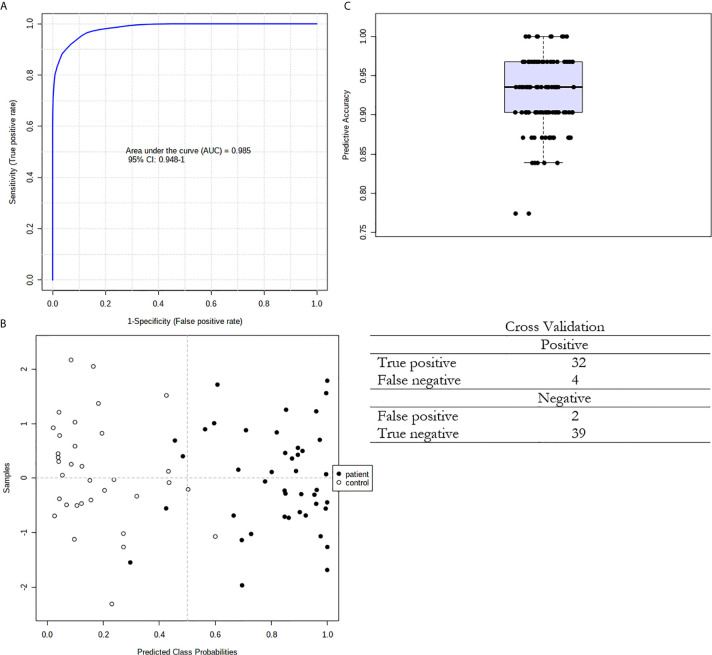
Multivariate ROC curve exploration. **(A)** Plot of the ROC curve for the created biomarker model based upon its average performance across all Monte Carlo cross-validation runs. The 95 percent confidence interval can be computed; **(B)** Plot of the predicted class probabilities for all samples using the created biomarker model. Due to balanced subsampling, the classification boundary is at the centre (x=0.5, dotted line); **(C)** Box plot of the predictive accuracy of the created biomarker model. The average accuracy based on 100 cross validations is 0.924.

We also used ROC curves to evaluate the performance of the potential biomarkers among selected 15 FAs using univariate and multivariate analyses. **Supplementary Figure S1** presents the ROC curves of the 15 selected FAs for cancer patients. The accuracy and other parameters of the ROC analysis of each FA are shown in **Table S2**. The results of this analysis showed that among the serum FAs, 11:0, 13:0, 14:0, 19:0, 14:1, CPOA2H, 12-M-14:0, 14-M-15:0 and 14-M-16:0 were the best predictors of CRC (AUC > 0.8). The multivariate ROC analysis using a panel consisting of the same FAs as those used in the univariate ROC analyses showed the best predictive ability with a ROC area of 0.985 for the tested dataset (**Figure 5**).

We also performed PCA of the whole FA profile in the tissues of CRC patients. The analysis also showed only slight differentiation between normal and cancer tissue (**Supplementary Figure S2**), but we can see a group of cancer tissue samples that differ significantly from normal tissue samples in terms of the FA profile, which was mostly due to the elevations of the levels of some of the long-chain saturated, monounsaturated and polyunsaturated FAs. On the other hand, short-chain FAs and some of the branched compounds were present in larger amounts in normal tissues.

Upon analyzing the FA profiles in different stages of CRC development, the changes observed in the whole group of studied CRC subjects (**Table 2**) were also observed in particular stages of CRC (**Supplementary Table S3**). However, when comparing the FA profiles in cancer tissue from patients at each stage of CRC by analysis of variance (ANOVA), no statistically significant differences were found. Additionally, we did not find any differences between the FA profiles in cancer tissue samples from patients in different stages of CRC development *via* PCA (**Supplementary Figure S3**).

## Discussion

Our study has shown significant changes in the FA profiles in both the serum and tumor tissue of CRC patients. The different FA profiles in the serum of CRC patients compared to those in the serum of healthy subjects may constitute a potential diagnostic tool, whereas the differences in FA profiles between CRC tumor tissue and normal colon mucosa may enable the identification of the molecular mechanisms underlying the changes seen in the serum. The most notable modification in the serum FA profile of patients with CRC is the presence of the VLCFAs 26:0, 28:0 and 26:1, which are absent in the serum of healthy subjects (28:0 and 26:1) or present in only trace amounts (26:0). This finding partially overlaps with that of our earlier study (6), which concerned the presence of 26:0 in the serum of CRC patients, but in this study, it was confirmed in a larger group of patients, and additional FAs (28:0 and 26:1) that are specifically found in the serum of CRC patients were identified. Moreover, in the present work, we attempted to discover the molecular mechanism underlying the occurrence of these specific VLCFAs in the blood of CRC patients. First, on the basis of the analysis of the FA profile in the cancer tissue of CRC patients and their normal colon mucosa, we found that the VLCFAs 26:0, 28:0 and 26:1 were the most highly increased FAs in cancer tissue compared to normal colon mucosa and were increased by 5-, 4.5- and 10-fold, respectively. We must bear in mind that the tumor constitutes a very small part of the CRC patient body. However, such a great increase in the content of the abovementioned VLCFAs may lead to an increase in their content in the blood, but this will only be the case if CRC cells are able to release these FAs into the bloodstream. The hypothesis of the CRC tumor origin of 26:0, 28:0 and 26:1 VLCFAs in the serum of CRC patients is also supported by our earlier study that showed the high overexpression of elongases ELOVL1 and ELOVL6 (elongation of very long chain fatty acids protein 1 and 6) in CRC tumors, which are responsible for the elongation of SFAs and MUFAs (6). However, these data allow us to speculate only on the mechanism of the occurrence of these VLCFAs in the serum of CRC patients. Thus, to experimentally verify our hypothesis, we used an *in vitro* model of HT-29 CRC cells. This experiment with ^13^C-labelled stearate allowed us to track its conversion in CRC cells. The results of this experiment are summarized in **Figure 1**, and they proved that a) ^13^C-18:0 enters CRC cells, b) ^13^C-18:0 in CRC cells is transformed into ^13^C-18:1 and ^13^C VLCFAs and c) VLCFAs produced from ^13^C-18:0 are released outside the cells. Thus, this *in vitro* experiment supports the hypothesis that CRC cells can produce and release VLCFAs into the blood and suggests that the VLCFAs 26:0, 28:0 and 26:1 in serum may be biomarkers of CRC. The question arises as to why CRC cells overproduce VLCFAs. A few studies have shown that elevated VLCFA in the cell membrane of cancer tissue compared to that of matched normal tissue is a common feature in various cancers (21). VLCFAs included in phospholipids and sphingolipids affect cell membrane structure and dynamics, which have an impact on cell size, division and differentiation as well as the membrane curvature (22). The long chain length of VLCFAs allows them to be simultaneously present in both leaflets of the lipid bilayer in the cell membrane, which stabilizes its curved shape (23). Moreover, the longer aliphatic chain of FA causes cell membranes to become more impervious (24). Knockdown of ELOVL1 increased apoptosis induced by anticancer drugs or UV (25). Thus, increased VLCFA synthesis in cancer cells may be a kind of protection against environmental factors that may cause cells to become “armored”.

Other FAs that are significantly changed in the serum of CRC patients are those from the BCFA group. These FAs originate from food and intestinal bacteria and can probably be produced in human adipose tissue (26). According to Vlaeminck et al. (27) BCFAs display anticancer properties, so the decrease in their levels may promote cancer development. We also found increase in 18:1 in serum of CRC patients. Since 18:1 is the major component of TAG (28), this change may be related to the increased TAG concentration in CRC patients. However, based on the results of our *in vitro* experiment (see **Figure 1**) and the increased expression of stearoyl-CoA desaturase-1 (SCD1) in CRC tissue (29), the release of 18:1 from CRC cells may also contribute to its elevated level in serum. In contrast, in cancer tissue, 18:1 was observed in smaller amounts than those in normal mucosa. Our recent study suggests that this may be due to the high energy requirement of cancer cells during their rapid proliferation; 18:1, which is found in TAG, undergoes intensive β-oxidation as an energy source in CRC cells (9). We also found lower levels of ALA and a trend toward a decrease in LA. As we reported in our earlier paper (14), decreased levels of these essential FAs may result from preferential uptake by cancer cells. The increased expression of the enzymes involved in PUFA elongation (ELOVL2, -4, and -5) and desaturation (fatty acid desaturases FADS-1 and -2) in cancer tissue compared to those in normal mucosa suggests that ALA and LA are further metabolized into longer and more highly unsaturated PUFAs in cancer tissue (14). PUFAs are essential for the formation of cell membrane phospholipids during the rapid proliferation of cancer cells (29). The rapid proliferation of cells is responsible for their smaller size and the higher percentage of cell membrane content compared to cytosol content (30). Lipids are responsible for at least 50% of the total membrane mass (31). This explains why in the present study, we found much higher SFA and PUFA levels (FAs that form cell membranes) in cancer tissue than in normal colon mucosa (**Table 4**).

As described above, our study suggests that 26:0, 28:0 and 26:1 may constitute potential tools for the non-invasive diagnosis of CRC. However, a good diagnostic tool should comprise as many parameters as possible. The method of FA profile analysis allows for the detection of over 40 different FAs in one analysis of human serum. Despite that multivariate PCA analysis of the whole FA profile in serum revealed that CRC patients and control subjects were not fully separated, a few statistical analyses using only the 15 FAs that were significantly different in serum between CRC patients and control subjects showed that these 15 FA may be considered as potential CRC biomarker. First, two-way hierarchical cluster analysis showed that this set of 15 FAs allowed us to separately cluster the controls and CRC patients. Second, both groups were well differentiated using advanced discrimination analysis (PLS-DA). Finally, the multivariate ROC curve analysis confirmed that this set of 15 FAs met the criteria of a very good diagnostic marker (AUC = 0.985). Moreover, our method allows us to combine this set of 15 FAs with 26:0, 28:0 and 26:1 in one GC-MS analysis. This indicates the particular value of our research; the good performance of the discriminant model presented here implies that CRC patients and healthy subjects may present with different lipidomic fingerprints. We discerned that these alterations in the evaluated FAs are due to the development of colorectal cancer.

The comparison of the FA profiles in cancer tissue and normal mucosa revealed that the contents of almost all FAs were different. These results indicate the complete alteration of the cancer tissue FA profile compared to that in normal mucosa. Unexpectedly, PCA of the FA profile in cancer tissue and normal mucosa did not show an unequivocal separation between the FA profiles in these two types of tissue. The possible reason for this apparent discrepancy is the high heterogeneity of the tumors and individual diversity. Most importantly, the results presented in **Table 4** were analyzed using a paired t-test, which means that the normal mucosa of every one of our 92 patients served as a control for his/her cancer tissue. PCA, as an unsupervised technique, does not allow us to take into account that each sample of cancer tissue and normal mucosa was obtained from the same subject, so perhaps this type of analysis is not very good for paired samples. Indeed, some authors did not find significant differences in the lipidome composition in cancer tissue and normal colonic mucosa using PCA (32). However, different lipid profiles in patients with locally advanced, unrespectable or metastatic colorectal cancer compared with those in healthy volunteers were found by PCA by de Figueiredo Junior et al. (31). We also did not find significant differences between individual FAs (analyzed by ANOVA – **Supplementary Table S3**) and the whole FA profile (analyzed by PCA) in cancer tissue from patients in successive stages of CRC, which was possibly due to the reasons discussed above.

In conclusion our study revealed the partial alteration of the FA profile in the serum of CRC patients and the complete alteration of the cancer tissue FA profile compared to that in normal mucosa. We have confirmed in *in vitro* experiment that CRC cells are able to synthesize and release VLCFAs, that may serve as potential noninvasive CRC biomarker. These results indicate that neoplastic transformation of intestinal cells causes significant changes in FA metabolism. Moreover, these disorders can also affect the composition of FA in the blood. We have also defined of a panel of FAs that may be assayed in a single analysis together with abovementioned VLCFAs and can serve as a potential reliable diagnostic marker indicating the presence of CRC and the need for a colonoscopy. However, our results need to be confirmed in a larger group of CRC patients and healthy controls prior to introducing this new diagnostic approach for use in the clinic.

## Data Availability Statement

The datasets presented in this study can be found in the article and in the **Supplementary Material**.

## Ethics Statement

The studies involving human participants were reviewed and approved by Local Bioethics Committee at the Medical University of Gdansk (decision no. NKBN/487/2015). The patients/participants provided their written informed consent to participate in this study.

## Author Contributions

AM, JK, and TS conceived the study. AM, KD, AP, and AC performed the experiments. AM, AP, and LH performed the statistical analyses. OR, MD, and JK treated the patients, and collected material and analyzed clinical data. AM, KD, AC, JK, and TS wrote the manuscript and all the authors assisted with editing the manuscript. All authors contributed to the article and approved the submitted version.

## Funding

This research was funded by National Science Centre of Poland, grant number 2016/22/E/NZ4/00665 and Medical University of Gdansk, grants number ST-40 and ST-89.

## Conflict of Interest

The authors declare that the research was conducted in the absence of any commercial or financial relationships that could be construed as a potential conflict of interest.
