# Supplementary material for: Rearrangements of Blood and Tissue Fatty Acid Profile in Colorectal Cancer - Molecular Mechanism and Diagnostic Potential
Source: Front Oncol. 2021 May 27;11:689701. doi: 10.3389/fonc.2021.689701 (PMC8190393; doi:10.3389/fonc.2021.689701)
Supplement: Supplementary file 1 [file DataSheet_1.docx]

Supplementary Material

Supplementary Table S1. Average coordinates of individuals from healthy control and CRC patients on the principal components 1-4 from PCA analysis based on the serum fatty acid profiles.

| **Principal component** | **% Variance explained** | **Healthy control** | **CRC patients** | **p (t-test)** |
| --- | --- | --- | --- | --- |
| PC1 | 23.40 | 2.73 ± 2.46 | -2.28 ± 2.90 | < 0.001 |
| PC2 | 14.06 | 0.93 ± 2.91 | -0.78 ± 2.59 | 0.009 |
| PC3 | 8.41 | -0.82 ± 1.14 | 0.68 ± 2.63 | 0.001 |
| PC4 | 6.14 | 0.37 ± 2.12 | -0.31 ± 1.63 | 0.127 |

Statistical significance of differences between both groups is also given (unpaired t-test, p-value).

Supplementary Table S2. Classical ROC curve analysis for univariate biomarker analysis.

| **Marker** | **AUC** | **T-tests** | **Log2 FC** | **Cluster** |
| --- | --- | --- | --- | --- |
| tridecanoic acid (13:0) | 0.93 | ˂0.001 | 11.07 | 4 |
| 9-tetradecenoic acid (14:1) | 0.86 | ˂0.001 | 8.15 | 3 |
| 14-methyl hexadecanoic acid (14-M-16:0) | 0.86 | ˂0.001 | 7.91 | 4 |
| CPOA2H | 0.86 | ˂0.001 | 8.04 | 4 |
| undecanoic acid (11:0) | 0.85 | ˂0.001 | 8.56 | 5 |
| 14-methyl pentadecanoic acid (14-M-15:0) | 0.81 | 0.009 | 6.09 | 4 |
| 12-methyl tetradecanoic acid (12-M-14:0) | 0.81 | ˂0.001 | 6.78 | 4 |
| myristic acid (14:0) | 0.81 | ˂0.001 | 6.78 | 3 |
| nondecanoic acid (19:0) | 0.81 | ˂0.001 | 6.95 | 5 |
| 13-methyl tetradecanoic acid (13-M-14:0) | 0.80 | ˂0.001 | 6.79 | 4 |
| lauric acid (12:0) | 0.78 | ˂0.001 | 5.39 | 3 |
| 13-docosenoic acid (22:1) | 0.77 | ˂0.001 | 5.51 | 3 |
| alfa linolenic acid (ALA) | 0.73 | 0.004 | 4.70 | 5 |
| oleic acid (18:1) | 0.72 | ˂0.001 | -4.28 | 2 |
| docosahexaenoic acid (DHA) | 0.69 | 0.019 | -3.82 | 1 |

Supplementary Table S3 Profile of fatty acids (%) in normal mucosa and CRC tissues in different stages of CRC.

|  | **UICC STAGE I n=18** | | | **UICC STAGE II n=27** | | | **UICC STAGE III n=33** | | | **UICC STAGE IV n=14** | | |
| --- | --- | --- | --- | --- | --- | --- | --- | --- | --- | --- | --- | --- |
|  | **NORMAL** | **CANCER** | **p** | **NORMAL** | **CANCER** | **p** | **NORMAL** | **CANCER** | **p** | **NORMAL** | **CANCER** | **p** |
| **16:0** | 22.4 ± 0.46 | 21.5 ± 0.40 | 0.065 | 21.3 ± 0.41 | 20.7 ± 0.44 | 0.322 | 21.7 ± 0.30 | 20.1 ± 0.35 | ˂0.001 | 21.0 ± 0.49 | 20.2 ± 0.32 | 0.087 |
| **18:0** | 7.57 ± 0.67 | 12.0 ± 1.00 | 0.001 | 8.05 ± 0.54 | 12.2 ± 0.68 | ˂0.001 | 7.77 ± 0.45 | 12.0 ± 0.49 | ˂0.001 | 7.48 ± 0.79 | 11.3 ± 0.91 | 0.003 |
| **20:0** | 0.16 ± 0.021 | 0.25 ± 0.026 | 0.007 | 0.17 ± 0.014 | 0.29 ± 0.033 | 0.001 | 0.17 ± 0.013 | 0.28 ± 0.021 | ˂0.001 | 0.18 ± 0.030 | 0.30 ± 0.043 | 0.057 |
| **22:0** | 0.14 ± 0.025 | 0.27 ± 0.040 | 0.006 | 0.15 ± 0.019 | 0.30 ± 0.031 | ˂0.001 | 0.14 ± 0.019 | 0.26 ± 0.018 | ˂0.001 | 0.17 ± 0.034 | 0.26 ± 0.041 | 0.110 |
| **24:0** | 0.13 ± 0.030 | 0.36 ± 0.068 | 0.004 | 0.14 ± 0.020 | 0.40 ± 0.051 | ˂0.001 | 0.12 ± 0.017 | 0.40 ± 0.041 | ˂0.001 | 0.17 ± 0.046 | 0.36 ± 0.065 | 0.044 |
| **26:0** | 0.009 ± 0.002 | 0.036 ± 0.005 | ˂0.001 | 0.010 ± 0.002 | 0.048 ± 0.008 | ˂0.001 | 0.009 ± 0.001 | 0.067 ± 0.015 | 0.001 | 0.012 ± 0.004 | 0.045 ± 0.006 | ˂0.001 |
| **28:0** | 0.001 ± 0.000 | 0.005 ± 0.001 | 0.001 | 0.002 ± 0.000 | 0.010 ± 0.002 | ˂0.001 | 0.002 ± 0.000 | 0.012 ± 0.003 | 0.001 | 0.001 ± 0.000 | 0.006 ± 0.001 | 0.005 |
| **other ECFA** | 2.43 ± 0.24 | 1.68 ± 0.21 | 0.004 | 2.62 ± 0.17 | 1.99 ± 0.19 | 0.005 | 2.64 ± 0.14 | 2.03 ± 0.16 | 0.001 | 2.53 ± 0.22 | 2.09 ± 0.21 | 0.031 |
| **ECFA** | 32.8 ± 0.82 | 36.1 ± 1.15 | 0.022 | 32.4 ± 0.69 | 36.0 ± 0.67 | ˂0.001 | 32.4 ± 0.36 | 34.9 ± 0.42 | ˂0.001 | 31.6 ± 1.15 | 34.5 ± 1.03 | 0.038 |
| **OCFA** | 0.72 ± 0.039 | 0.92 ± 0.058 | 0.012 | 0.81 ± 0.060 | 0.94 ± 0.053 | 0.051 | 0.72 ± 0.030 | 0.83 ± 0.040 | 0.008 | 0.73 ± 0.065 | 0.86 ± 0.078 | 0.199 |
| **iso BCFA** | 0.23 ± 0.016 | 0.21 ± 0.014 | 0.129 | 0.25 ± 0.017 | 0.27 ± 0.022 | 0.189 | 0.23 ± 0.016 | 0.21 ± 0.010 | 0.057 | 0.20 ± 0.013 | 0.21 ± 0.015 | 0.386 |
| **anteiso BCFA** | 0.16 ± 0.015 | 0.15 ± 0.011 | 0.550 | 0.15 ± 0.011 | 0.17 ± 0.018 | 0.277 | 0.16 ± 0.015 | 0.14 ± 0.011 | 0.189 | 0.11 ± 0.012 | 0.14 ± 0.014 | 0.002 |
| **TOTAL SFA** | **33.9 ± 0.85** | **37.4 ± 1.18** | **0.019** | **33.6 ± 0.75** | **37.4 ± 0.70** | **˂0.001** | **33.5 ± 0.39** | **36.1 ± 0.44** | **˂0.001** | **32.6 ± 1.21** | **35.8 ± 1.08** | **0.037** |
| **16:1** | 4.32 ± 0.37 | 3.47 ± 0.26 | 0.035 | 4.54 ± 0.30 | 3.94 ± 0.28 | 0.111 | 4.77 ± 0.32 | 3.59 ± 0.18 | 0.001 | 4.43 ± 0.45 | 3.49 ± 0.26 | 0.053 |
| **18:1** | 43.4 ± 1.22 | 36.1 ± 1.44 | ˂0.001 | 42.5 ± 1.13 | 34.9 ± 1.32 | ˂0.001 | 43.0 ± 0.86 | 36.5 ± 0.95 | ˂0.001 | 43.0 ± 1.98 | 36.7 ± 2.05 | 0.009 |
| **20:1** | 0.63 ± 0.039 | 0.62 ± 0.051 | 0.806 | 0.64 ± 0.046 | 0.73 ± 0.079 | 0.268 | 0.64 ± 0.043 | 0.72 ± 0.048 | 0.184 | 0.60 ± 0.048 | 0.86 ± 0.070 | 0.007 |
| **22:1** | 0.054 ± 0.011 | 0.096 ± 0.014 | 0.003 | 0.051 ± 0.005 | 0.12 ± 0.019 | 0.001 | 0.058 ± 0.008 | 0.11 ± 0.010 | ˂0.001 | 0.054 ± 0.009 | 0.143 ± 0.016 | ˂0.001 |
| **24:1** | 0.15 ± 0.032 | 0.41 ± 0.10 | 0.025 | 0.15 ± 0.020 | 0.42 ± 0.06 | ˂0.001 | 0.13 ± 0.017 | 0.45 ± 0.04 | ˂0.001 | 0.20 ± 0.063 | 0.49 ± 0.10 | 0.020 |
| **26:1** | 0.001 ± 0.000 | 0.014 ± 0.002 | ˂0.001 | 0.002 ± 0.001 | 0.016 ± 0.002 | ˂0.001 | 0.002 ± 0.000 | 0.028 ± 0.006 | ˂0.001 | 0.002 ± 0.001 | 0.019 ± 0.004 | ˂0.001 |
| **other MUFA** | 0.41 ± 0.049 | 0.32 ± 0.028 | 0.056 | 0.41 ± 0.029 | 0.35 ± 0.032 | 0.156 | 0.43 ± 0.037 | 0.31 ± 0.025 | 0.001 | 0.34 ± 0.044 | 0.29 ± 0.024 | 0.130 |
| **Total MUFA** | **49.0 ± 1.46** | **41.0 ± 1.64** | **0.001** | **48.3 ± 1.37** | **40.5 ± 1.45** | **0.001** | **49.1 ± 1.10** | **41.7 ± 1.09** | **˂0.001** | **48.7 ± 2.24** | **42.0 ± 2.18** | **0.012** |
| **ALA** | 0.038 ± 0.007 | 0.046 ± 0.007 | 0.236 | 0.041 ± 0.004 | 0.056 ± 0.006 | 0.028 | 0.036 ± 0.003 | 0.043 ± 0.007 | 0.299 | 0.034 ± 0.003 | 0.043 ± 0.010 | 0.254 |
| **EPA** | 0.23 ± 0.040 | 0.45 ± 0.058 | 0.001 | 0.25 ± 0.035 | 0.42 ± 0.057 | 0.011 | 0.26 ± 0.036 | 0.45 ± 0.048 | ˂0.001 | 0.35 ± 0.062 | 0.52 ± 0.084 | 0.046 |
| **DHA** | 0.50 ± 0.062 | 0.98 ± 0.097 | ˂0.001 | 0.59 ± 0.064 | 1.03 ± 0.108 | ˂0.001 | 0.61 ± 0.055 | 1.17 ± 0.11 | ˂0.001 | 0.74 ± 0.12 | 1.08 ± 0.10 | 0.007 |
| **other PUFAn3** | 0.36 ± 0.018 | 0.59 ± 0.045 | 0.001 | 0.35 ± 0.032 | 0.60 ± 0.059 | 0.002 | 0.37 ± 0.021 | 0.66 ± 0.052 | ˂0.001 | 0.43 ± 0.049 | 0.66 ± 0.068 | 0.006 |
| **Total PUFAn3** | **1.13 ± 0.11** | **2.06 ± 0.17** | **˂0.001** | **1.24 ± 0.12** | **2.10 ± 0.19** | **˂0.001** | **1.27 ± 0.10** | **2.32 ± 0.19** | **˂0.001** | **1.55 ± 0.22** | **2.31 ± 0.21** | **0.002** |
| **LA** | 11.6 ± 0.38 | 11.2 ± 0.52 | 0.557 | 11.9 ± 0.42 | 11.3 ± 0.56 | 0.286 | 11.4 ± 0.42 | 10.8 ± 0.31 | 0.103 | 11.7 ± 0.39 | 10.6 ± 0.50 | 0.132 |
| **ARA** | 3.24 ± 0.52 | 6.00 ± 0.61 | 0.001 | 3.59 ± 0.48 | 6.42 ± 0.71 | 0.002 | 3.52 ± 0.42 | 6.59 ± 0.50 | ˂0.001 | 4.09 ± 0.74 | 6.80 ± 0.91 | 0.002 |
| **DGLA** | 0.41 ± 0.065 | 1.23 ± 0.13 | ˂0.001 | 0.37 ± 0.055 | 1.14 ± 0.12 | ˂0.001 | 0.30 ± 0.038 | 1.22 ± 0.10 | ˂0.001 | 0.34 ± 0.061 | 1.12 ± 0.14 | ˂0.001 |
| **AdA** | 0.39 ± 0.042 | 0.71 ± 0.10 | 0.007 | 0.39 ± 0.033 | 0.80 ± 0.12 | 0.002 | 0.39 ± 0.027 | 0.86 ± 0.07 | ˂0.001 | 0.47 ± 0.069 | 0.98 ± 0.21 | 0.032 |
| **other PUFAn6** | 0.35 ± 0.039 | 0.41 ± 0.036 | 0.390 | 0.61 ± 0.11 | 0.37 ± 0.038 | 0.071 | 0.45 ± 0.047 | 0.40 ± 0.027 | 0.391 | 0.55 ± 0.11 | 0.45 ± 0.043 | 0.431 |
| **Total PUFAn6** | **16.0 ± 0.77** | **19.6 ± 1.04** | **0.006** | **16.9 ± 0.88** | **20.0 ± 1.05** | **0.021** | **16.1 ± 0.84** | **19.8 ± 0.69** | **˂0.001** | **17.2 ± 1.05** | **20.0 ± 1.10** | **0.007** |

Values are mean ± SEM. AdA – adrenic acid (22:4 n-6); ALA – α-linolenic acid (18:3 n-3); ARA – arachidonic acid (20:4 n-6); BCFA – branched chain fatty acids; DGLA –dihomo-γ-linolenic acid (20:3 n-6); DHA – docosahexaenoic acid (22:6 n-3); ECFA – even chain fatty acids; EPA – eicosapentaenoic acid (20:5 n-3); LA – linoleic acid (18:2 n-6); MUFA – monounsaturated fatty acids, OCFA – odd chain fatty acids; PUFA – polyunsaturated fatty acids; SFA – saturated fatty acids. Boldface - major groups of fatty acid.


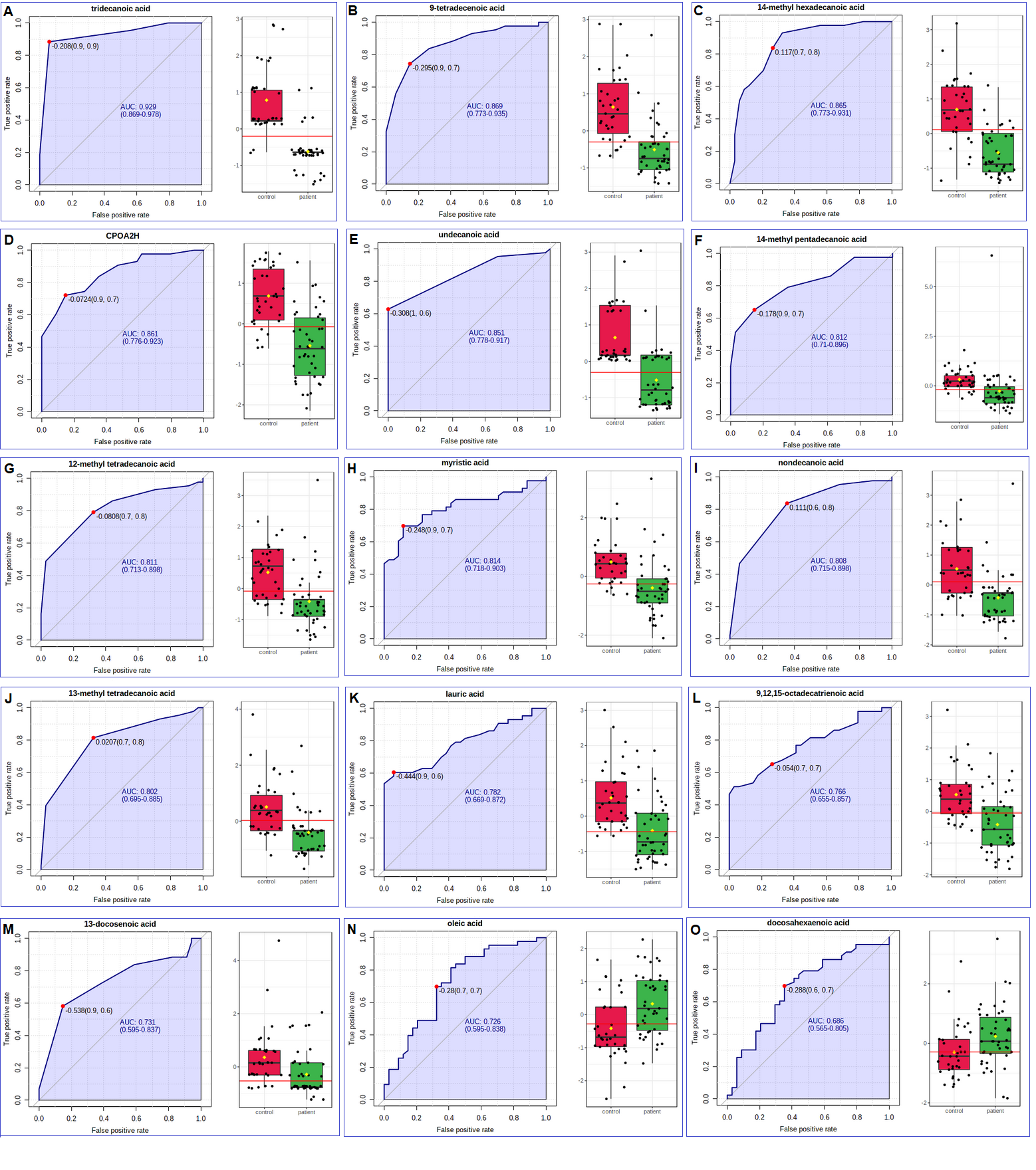
Supplementary Figure S1 The ROC curves and box-plot of the contents of the selected FA between two groups within the dataset. In ROC Curves Analysis the sensitivity is on the y-axis, and the specificity is on the x-axis. The area-under-the-curve (AUC) is in blue. On box-plot horizontal line is in red indicating the optimal cutoff.

**
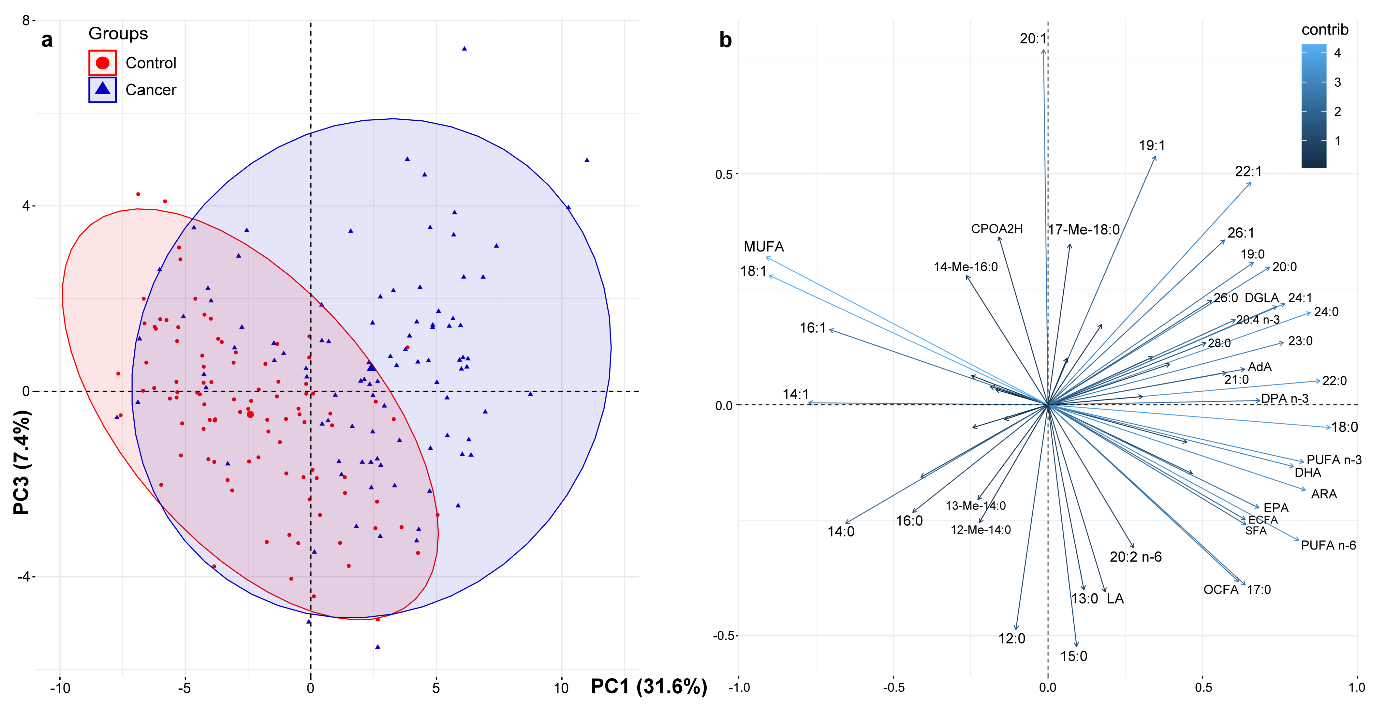
**

Supplementary Figure S2. The results of principal component analysis (PCA) based on the whole fatty acids profile in tissues of CRC patients: score plots of cases (a) and variables (b). Please consult Table 1 for abbreviations and statistical significance of differences between amounts of compounds in healthy and cancer tissues; some minor variables were not shown to keep the clarity of presentation.


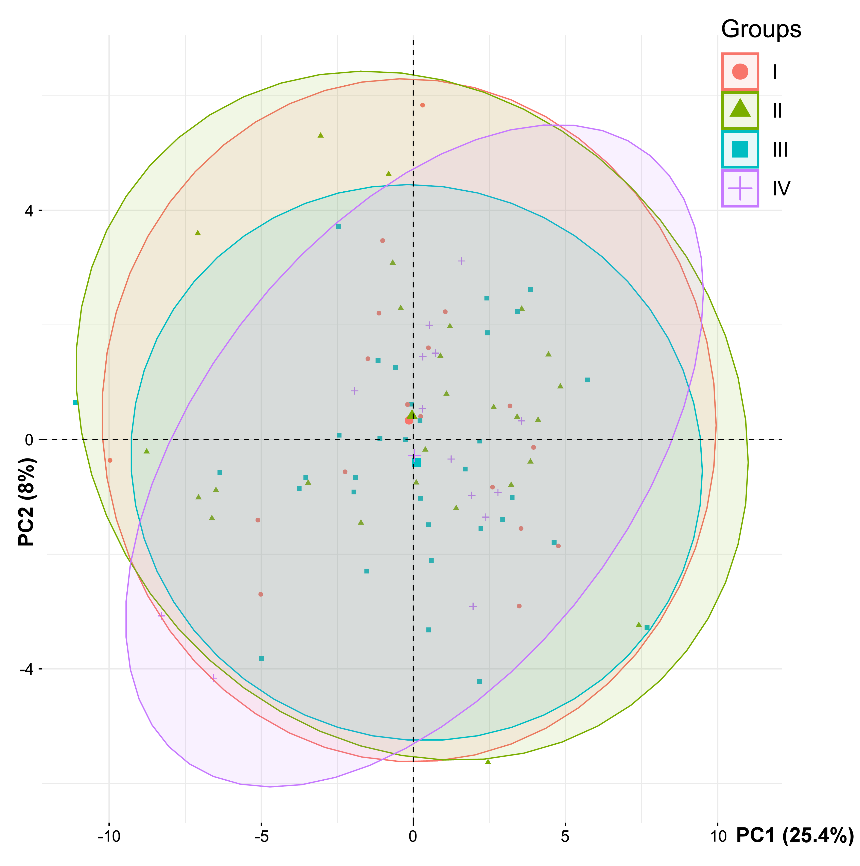


Supplementary Figure. S3. The results of principal component analysis (PCA) of individuals based on the whole fatty acids profile in cancer tissue in particular stages of CRC.
